# Repeated 5-aminolevulinic acid mediated sonodynamic therapy using magnetic resonance guided focused ultrasound in rat brain tumour models

**DOI:** 10.1038/s41598-025-85314-6

**Published:** 2025-01-07

**Authors:** Sheng-Kai Wu, Chia-Lin Tsai, Aisha Mir, Stuart L. Marcus, Kullervo Hynynen

**Affiliations:** 1 Physical Sciences Platform, Sunnybrook Research Institute, Toronto, ON Canada; 2 Department of Medical Biophysics, University of Toronto, Toronto, ON Canada; 3 Department of Neurology, National Defense Medical Center, Tri-Service General Hospital, Taipei, Taiwan; 4 SonALAsense Inc, Oakland, CA USA; 5 Institute of Biomaterials and Biomedical Engineering, University of Toronto, Toronto, ON Canada

**Keywords:** Sonodynamic therapy, 5-Aminolevulinic acid, Brain tumour, MR-guided focused ultrasound, Burst length, Neuroscience, Neurology, Oncology, Cancer therapy, Targeted therapies, Biomedical engineering

## Abstract

Sonodynamic therapy is an emerging therapeutic approach against brain tumours. However, the treatment scheme and ultrasound parameters have yet to be explored for clinical translation. Our study aimed to optimize ultrasound parameters for sonodynamic therapy (SDT) with 5-ALA as a sonosensitizing agent and to evaluate its therapeutic outcome on the rodent 9L gliosarcoma and the human U87 glioblastoma models. We stereotactically implanted brain tumour cells in rats and monitored tumour volume via MRI. SDT was conducted weekly using a 60 mg/kg dose of 5-ALA, injected intravenously 6 h before sonication. We used a driving frequency of 580 kHz with 0.75 MPa and evaluated the effect of different burst lengths to optimize ultrasound parameters. We also tested SDT against advanced-stage brain tumours to verify its efficacy further. Our results showed that a longer burst length could improve therapeutic outcomes. Tumour growth inhibition was established only in the first three weeks with 10 ms and 50 ms burst length sonication, but 86 ms burst length greatly improved the survival outcome. Therefore, the therapeutic efficacy is proportionate to the burst length and, thus, the total delivered energy. Repeated SDT using multiple targets to cover the entire tumour volume with optimal ultrasound parameters can achieve significant anti-tumour effects in both 9L and U87 models. Lastly, our results on late-stage tumour treatments showed that SDT can still provide prolonged survival. These promising findings demonstrate that repeated SDT using transcranial-focused ultrasound together with 5-ALA can optimize anti-tumour effects and even lead to complete clearance of the tumours. This weekly treatment with pulsed ultrasound sonication strategy is practical for future clinical translation.

## Introduction

Glioblastoma multiforme (GBM) is categorized as a highly aggressive cancer of the central nervous system (CNS) by the World Health Organization (WHO) and has a dismal prognosis^1,2^. Therefore, multiple treatment modalities have been combined, including surgical resection, radiotherapy, and chemotherapy^3^. Furthermore, several chemotherapeutic agents were developed to treat GBM, such as temozolomide (TMZ)^4^, bevacizumab^5^, cisplatin^6^, carmustine (BCNU)^7^, etc. To date, the most active chemotherapeutic agent, TMZ, combined with surgery and radiotherapy, can only prolong median survival by 2.5 months^4^, and recurrence is still unavoidable^8^. Hence, new therapeutic regimens for glioblastoma are sorely needed.

5-ALA Sonodynamic therapy (SDT) is an innovative therapeutic approach to treat cancers. It utilizes ultrasound energy to activate the sonosensitizing agent, protoporphyrin IX (PpIX), a metabolite of 5-ALA accumulated within malignant gliomas. SDT produces a cytotoxic effect only within tumours in the targeted region via a photodynamic therapy (PDT) effect^9,10^. SDT has demonstrated promising results in preclinical animal glioma models^9,11,12^, and multiple clinical trials of 5-ALA SDT in human malignant glioma patients are ongoing^13–15^. During 5-ALA SDT, 5-ALA is administered through intravenous injection or oral consumption. Once PpIX has accumulated preferentially in the tumour tissue, the ultrasound waves are subsequently applied to the targets. Ultrasound exposure can activate PpIX, producing reactive oxygen species (ROS) and causing cytotoxicity. ROS produced by SDT oxidizes cellular components such as plasma and mitochondrial membranes, leading to cell death via apoptosis and other mechanisms^16^. SDT has advantages over PDT in treating deep-seated tumours due to the deeper penetration of ultrasound when compared with light^17^. In addition, SDT can also promote anti-inflammatory and immunomodulatory responses^18,19^.

5-ALA and fluorescein have been investigated for their potential to serve as sonosensitizers in SDT. 5-ALA is the first committed metabolite of the heme pathway. In heme biosynthesis, the last step is the addition of an iron atom to PpIX by ferrochelatase (FECH), located in the inner membrane of mitochondria. Dysfunction of the FECH might be a potential mechanism that leads to PpIX accumulation in tumours^20–22^. Silencing of FECH has been proven to enhance 5-ALA-based PDT in glioma cells due to increasing the accumulation of PpIX. In fact, FECH is decreased in prostate cancer, bladder cancer, and colonic tumour^23–25^. The accumulation of PpIX in malignant brain tumours after exposure to exogenous excess 5-ALA is used every day as a visual aid for neurosurgical resections^26–29^. The route of 5-ALA administration is either oral or intravenous. The temporal kinetics of both routes are similar; however, a higher oral dose is required to achieve the same tissue accumulation of PpIX^30^. The peak fluorescence intensity occurs in tumours around four to eight hours after 5-ALA administration^31^ providing a time window for ultrasound exposure. Although many previous studies have investigated the frequency range of around 1 MHz for SDT, frequency as low as 25 kHz has shown the ability to achieve the SDT therapeutic effect in an in vivo U87-MG glioma model^32^. A clinically relevant frequency of 220 kHz was combined with 5-ALA in a brain tumour model and showed the feasibility of tumour suppression and minimal damage to the normal healthy brain tissue^33^.

Previously, we have shown the superiority of SDT in C6 glioma treatment in vivo and the improvement of survival^12^ and that different core temperature baselines do not significantly influence the therapeutic outcome of SDT. Unlike surgical resection of brain tumours, SDT can be applied repeatedly without physical access to the brain. Here, we first demonstrate SDT in a weekly treatment scheme in rodent 9L gliosarcoma and human U87 MG brain tumour models. Following treatment, the efficacy data was collected, as well as histopathological analysis. Although previous studies showed that continuous ultrasound sonication could yield therapeutic effects in brain tumour therapy^11,12^, pulsed ultrasound is a better approach to achieve transcranial ultrasound exposure without elevating the skull temperature and being able to cover a larger tumour volume. Thus, we pulsed the ultrasound waves in this study to avoid thermal deposition within the skull. In addition, we further investigate the effect of different burst lengths on the therapeutic outcome. Furthermore, we extended the treatment to late-stage tumours to confirm the potential benefits of SDT in enhancing therapeutic effectiveness in the advanced tumour stage.

## Results

### The impact of burst length under the same pressure level for the SDT efficacy on the 9L brain tumour model

**Fig. 1:**
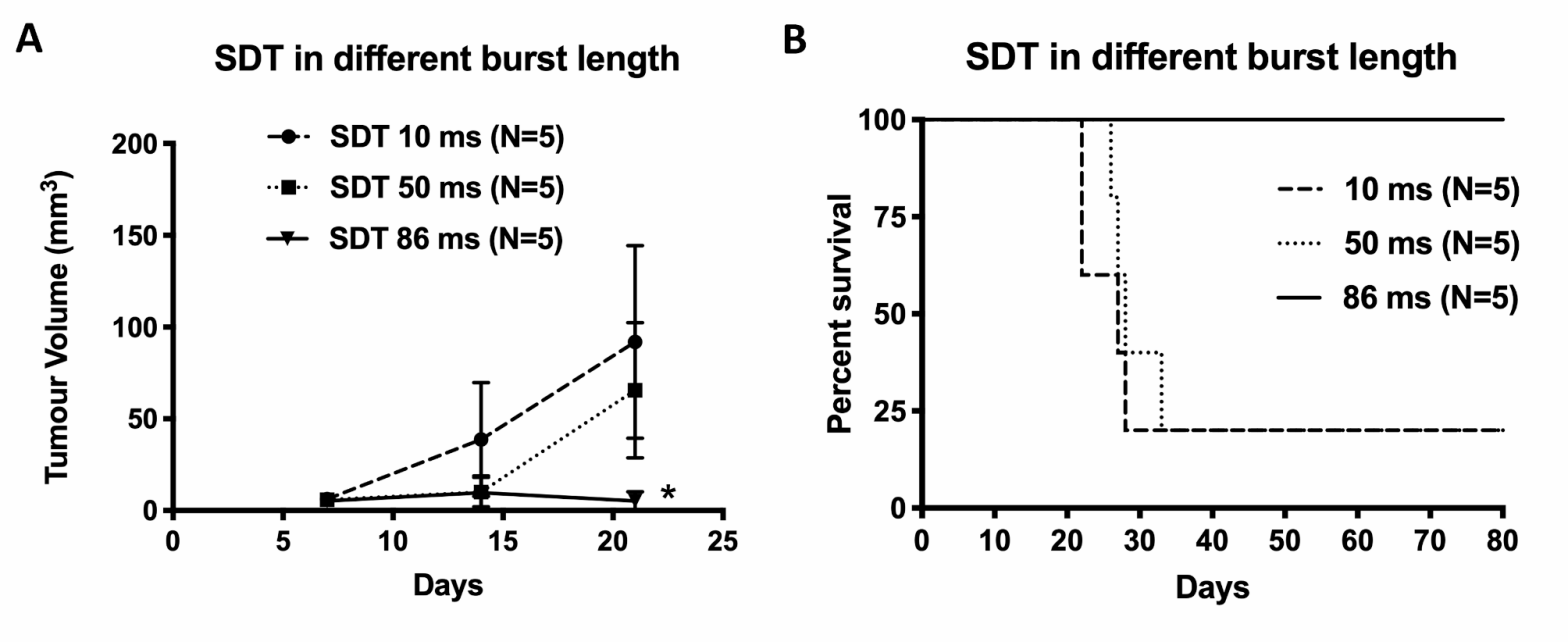
Three different burst lengths (10, 50, and 86 ms) were employed to treat the 9L brain tumour. (**A**) Three weeks of the shorter burst lengths (10 and 50 ms) treatment did not inhibit the tumour from growing as the 86 ms burst length. In addition, 50 ms burst length has slightly slowed the tumour growth, but no difference was found between these two parameters. (**B**) No significant difference in survival was detected between 10 and 50 ms. In contrast, 86 ms burst length resulted in a 100% survival rate.

In Fig. 1A, the tumour growth with the different burst lengths is shown and demonstrates that the 86 ms exposure with the 300 bursts/location and three treatments was effective in completely inhibiting the tumour growth. In addition, the survival data (Fig. 1B) also showed a median survival time of 27 days for the 10 ms group and 28 days for the 50 ms group but a prolonged survival for the 86ms exposures. This indicated that 10 ms or 50 ms burst length with 300 burst/ location had a limited therapeutic impact on the 9L brain tumour model.

### Repeated sonodynamic therapies for the 9L brain tumour model

**Fig. 2:**
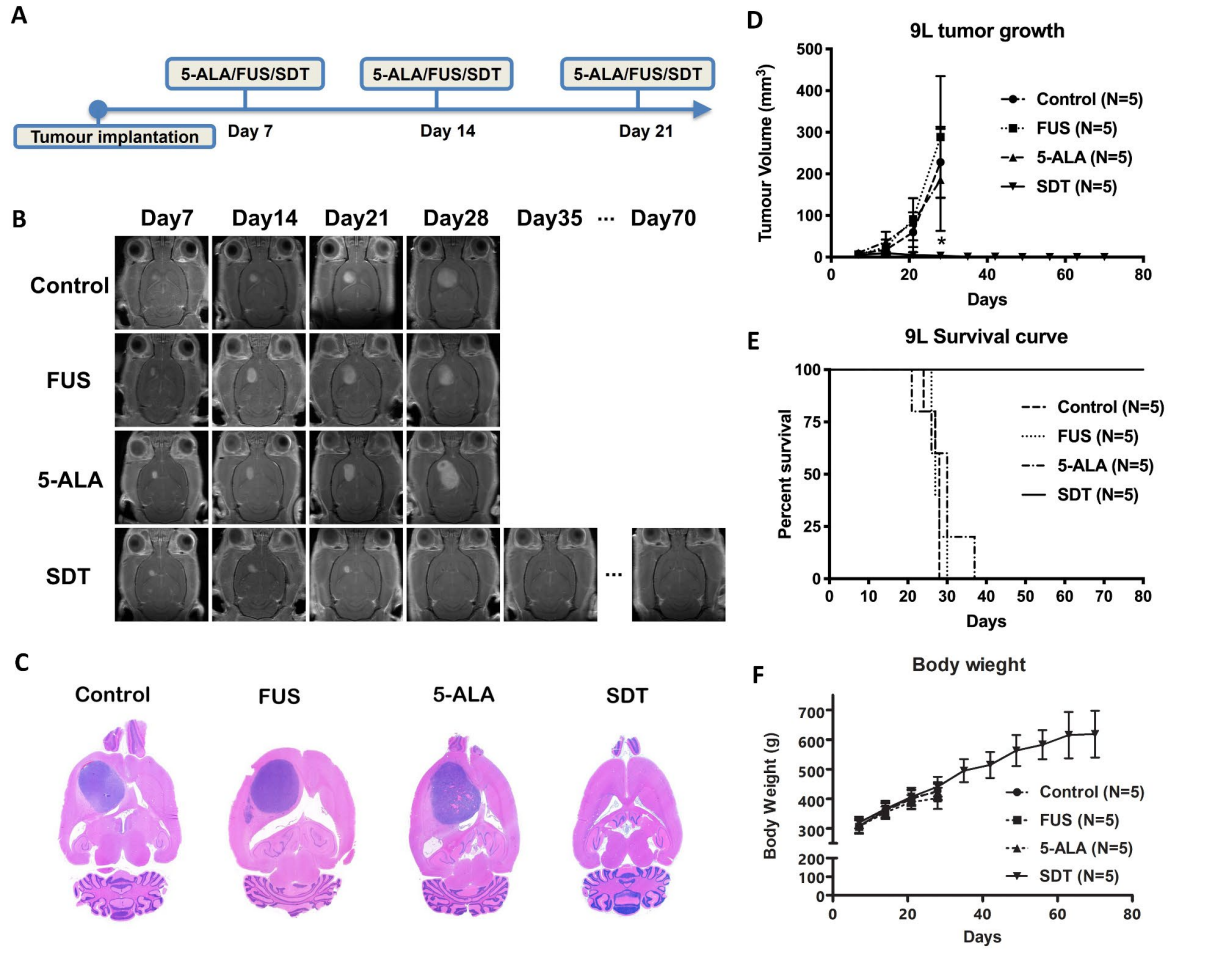
(**A**) For the 9L brain tumour treatment, the treatments started seven days after the tumour implantation, and the following treatments were conducted at 14 and 21 days. (**B**) The representative T1 contrast-enhanced (T1-CE) MR images of control, FUS alone, 5-ALA alone, and SDT treatment. At day 28, the 9L tumour is significantly shrunk. The surviving animals were followed up for 80 days. (**C**) The representative H&E stains of all groups. On day 80, the animals that underwent SDT were tumour-free. The rest showed an enormous brain tumour volume. (**D**) The tumour growth curve was analyzed using the One-way ANOVA with post-Dunn’s test. The significance can be found in the comparison between SDT and the other three groups. (**E**) Survival was analyzed using the log-rank test, and a p-value of 0.003 was shown. After three treatments, SDT showed a significant tumour reduction, and all the animals survived to the designated endpoint. (**F**) No difference in animal body weight was found among all groups during the three weeks of treatments, indicating the animals tolerated SDT well.

To determine whether focused ultrasound-facilitated sonodynamic agent activation can produce an anti-cancer effect on the 9L gliosarcoma rat model in a weekly repeated manner (Fig. 2A). The control tumours grew over the designed endpoint (tumour volume exceeded 200 mm^3^) in the third week and reached the endpoint (Fig. 2B and D). The rats in the SDT group were tumour-free after three weeks of SDT. In this brain tumour model, the median survival time for the control, FUS alone, and 5-ALA animals was 28, 27, and 30 days, respectively (Fig. 2E). In contrast, the SDT-treated animals showed a significantly prolonged survival time (80 days) (*p* < 0.01). Figure 2C demonstrates the H&E stain of the representative images of different treatments. In the SDT brain, a slightly enlarged ventricle on the tumour side of the brain can still be observed, but no solid tumour can be found in the rat’s right-side striatum. However, control, FUS, and 5-ALA rats showed a solid tumour occupied the right brain with distorted ventricles. In Fig. 2F, SDT animals gradually gained weight throughout the study, indicating they tolerated SDT well.

## Repeated sonodynamic therapies for the late-stage 9L brain tumour model

**Fig. 3:**
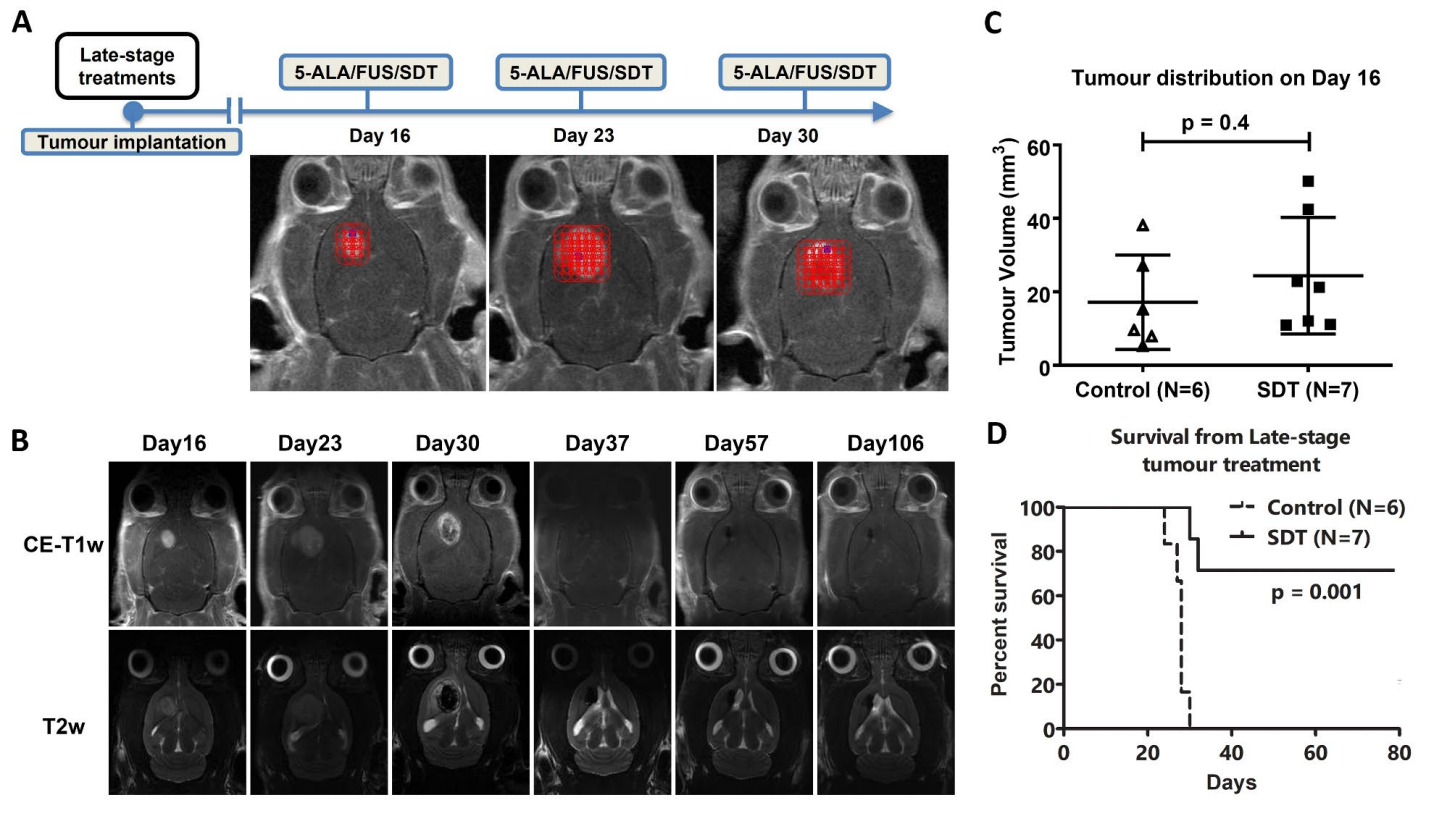
(**A**) To investigate late-stage tumours in response to SDT, the weekly therapy started from Day 16 and continued for the following two weeks. The FUS targets varied in correlation to the tumour sizes. (**B**) A solid tumour was shown in the right striatum on Day 16. After two treatments, a clear necrotic centre appeared in the T2w images on Day 30, indicating the efficiency of SDT. (**C**) The tumour volume comparison between Day 7 and Day 16 brain tumours indicates the significance of the late-stage tumour volume. (**D**) Kaplan-Meier survival analysis where groups were compared with a log-rank test and a Bonferroni correction for multiple comparisons; *p* = 0.002.

Despite a successful tumour inhibition of SDT starting from day 7, we further assess whether SDT can have a therapeutic impact on a late-stage tumour. Figure 3A demonstrates the procedure, where the treatments began on day 16 and the following two weeks. Figure 3C established the tumour size difference between days 7 and 16 (*p* < 0.01). The representative MR images are displayed in Fig. 3B. At day 30, a clearly necrotic center was formed in the treated area, and the tumour started to shrink after three sessions of SDT. The survival results (Fig. 3D) for the large tumour receiving repeated SDT showed a significant improvement compared to the control animals (*p* < 0.01). Five out of the seven animals showed a tumour-free response.

## Repeated sonodynamic therapies for the human U87 brain tumour model

**Fig. 4:**
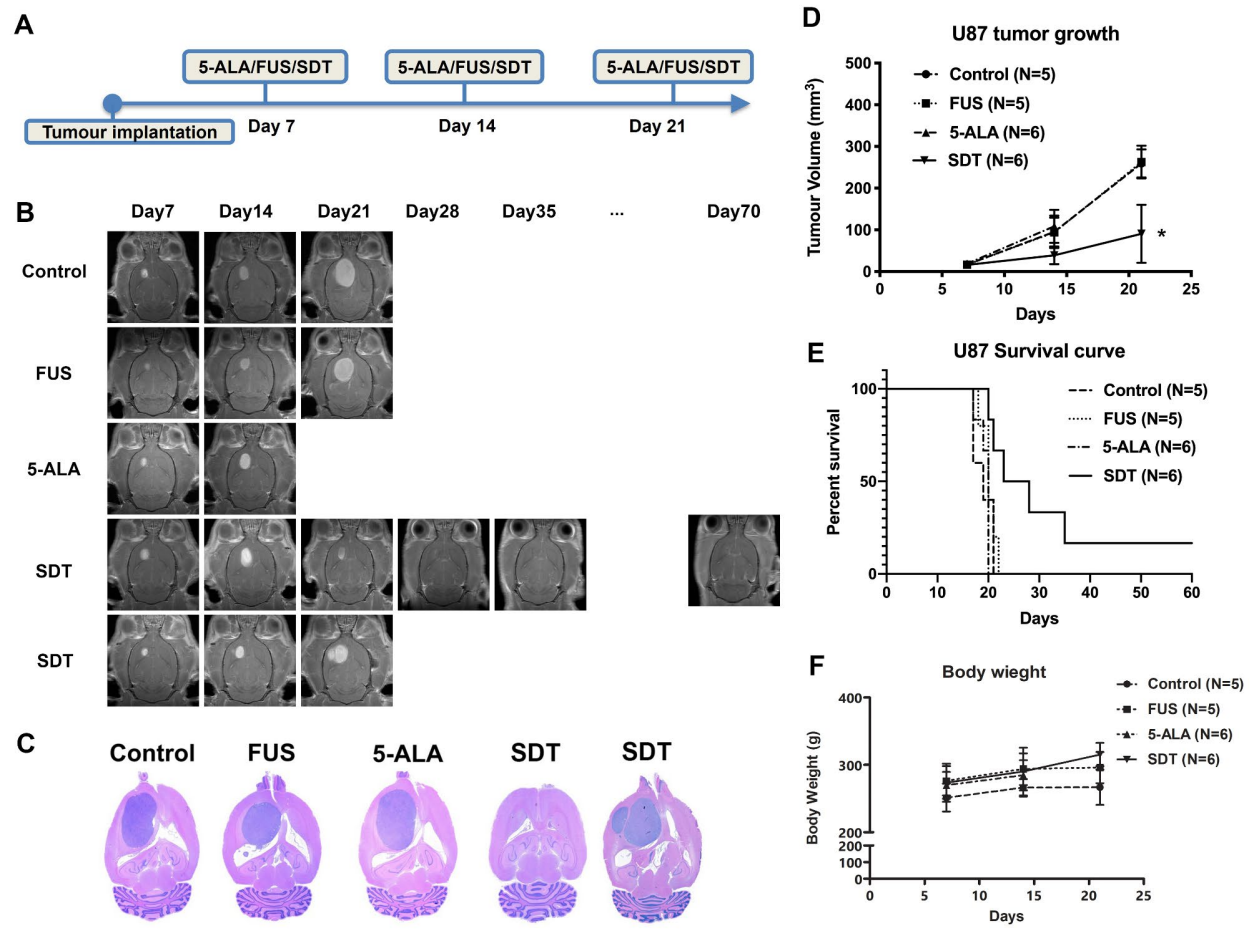
(**A**) We further investigate the efficiency of SDT in the human glioblastoma U87 rat model. The animal received three weekly treatments beginning from day 7. (**B**) The representative T1 contrast-enhanced MR images of control, FUS alone, 5-ALA alone, and SDT treatment. The first row in the SDT group demonstrated the long-term tumour-free survivor. The tumour disappeared after three treatments. However, SDT had a minimal impact on some animals, such as the second row in the SDT group. (**C**) The H&E stains stood for the animals from (**B**), respectively. (**D**) A significant difference was found in the third week between the control group and the SDT group. (**E**) The log-rank test showed prolonged survival in the SDT group, *p* = 0.01. (**F**) The body weight in all groups did not vary during three-week treatments.

Finally, we utilized the repeated SDT with 86 ms burst length on the immunocompromised rats with human U87 glioblastoma brain tumours. The treatment plan is shown in Fig. 4A. Figure 4B exhibits the representative MR images. Control, FUS alone, and 5-ALA alone group showed a rapid progression of tumour growth. However, SDT only slows down the tumour growth but does not inhibit the tumour from progression. In Fig. 4C, solid tumours occupied the right side of the rat brain in control, FUS alone, and 5-ALA alone groups at approximately day 20. Here, we demonstrated the tumour-free rat in the SDT group and one of the SDT animals that reached the endpoint at day 28. Hence, the difference in tumour growth (Fig. 4D) can only be detected at the third treatment (day 21). In the SDT-treated group, one animal had a very positive response to SDT, and the tumour was not visible after three sessions of SDT. In Fig. 4E, the median survival time for control, FUS alone, 5-ALA alone, and SDT are 19, 20, 20, and 25.5 days, respectively (*p* < 0.01). Figure 4F indicates that immunocompromised rats can tolerate SDT well.

## Discussion

In this study, we conducted a series of comprehensive experiments to investigate the efficacy of ultrasound and 5-ALA as sonosensitizing agents for SDT of rodent brain tumours. In the 9L gliosarcoma rodent brain tumour model, we found that three weekly treatments yielded promising therapeutic efficacy and completely eliminated the brain tumour, prolonging the animal’s survival with the optimal ultrasound parameters. The sonodynamic effect relies on the combined use of a sonodynamic agent and ultrasound sonication to effectively target tumours; neither can function independently. This study demonstrates that neither focused ultrasound (FUS) alone nor 5-ALA by itself produced significant anti-tumour effects when compared to the control group in rodent 9L and human U87 tumour models. As the tumours progressed, the body weight of the tumour-bearing animals began to plateau. In contrast, the animals that received SDT consistently gained weight over time. This could be attributed to both the regression of the brain’s tumours and the SDT treatment’s tolerability. Notably, the normal brain tissue surrounding the tumour area did not exhibit any significant changes in the MRI images.

A previous study showed that continuous wave ultrasound sonication at the frequency of 1 MHz is effective in inhibiting rodent C6 glioma tumours^12^. Still, it could slightly increase the tissue temperature and cause inevitable skull heating. Therefore, in this study, we used a frequency of 500 kHz and a burst length of 86 ms with a pressure of 0.75 MPa, which yielded therapeutic effects against rodent 9L brain tumours. These exposures can be safely delivered through the human skull, making therapy clinically feasible. Pulsed, low-frequency ultrasound exposures will reduce thermal accumulation in the skull and make it feasible to cover a larger tumour in a reasonable time. In this study, we also tried the burst lengths of 10 and 50 ms, but we observed a reduction in the therapeutic outcome compared to the 86 ms burst length with the selected 300 bursts/location with 0.75 MPa. This means that the sonosensitizer must interact with ultrasound for a minimum period to have cytotoxic effects. Therefore, treatment impact may depend on the total energy delivered into each sonication location, and it may be possible that a higher-pressure amplitude exposure or increased burst number could have been effective with these shorter exposures. We were not able to study the higher-pressure exposures in this study due to experimental limitations.

Although we have shown the effectiveness of 5-ALA SDT using pulsed ultrasound, the burst length used in this study may still cause significant standing waves in the small rat skull cavity. This can lead to high variability in the actual pressure amplitude experienced by the tumor tissue. Therefore, our estimates of the actual acoustic pressure may not be precise and directly applicable to patient treatments^34^. Moreover, the activation of 5-ALA may be different when the standing waves are eliminated, as is the case in a larger skull cavity.

SDT that uses 5-ALA has been tested in various parameters, including ultrasound operating frequency. Some studies have used a significantly lower frequency of 25 kHz to treat U87-MG glioma in nude mice subcutaneous model^32^, while others have reported that SDT is promising at a higher frequency of 2.2 MHz^35^. These studies, combined with this study’s results, indicate that the SDT effect could be achieved over a wide ultrasound frequency range. Based on our burst length results, it seems that SDT might be influenced by energy levels. Under consistent pressure, longer bursts yield a more significant anti-tumor effect. Theoretically, a burst longer than 86 ms could have a greater impact on U87 tumours than an 86 ms burst. However, prolonged bursts may not be feasible in clinical settings, as they could lead to increased skull heating. Therefore, a more detailed investigation of ultrasound parameters is necessary to address this issue. With the current single-element setting, the higher-pressure sonication was observed to cause pre-focal cavitation at the scalp interface. However, a phased array system can overcome this limitation.

The generation of reactive oxygen species (ROS) induced by SDT may play a crucial role in its mechanism. Measuring the levels of ROS during treatment could enhance our understanding of how SDT works. In clinical settings, 5-ALA is utilized for image-assisted tumour resection due to its fluorescence properties. Therefore, direct measurement of ROS production during SDT could be performed using a fluorescence imaging system integrated with the ultrasound system, such as two-photon microscopy paired with a ring-shaped ultrasound transducer setup^36^. In the rat brain tumour model 9L, SDT was found to slow down tumour growth and prolong survival by causing programmed cell death in tumour cells through the release of ROS by ultrasound-triggered sonosensitive agents^37,38^. However, in the human U87 rat brain tumour model, SDT did not produce the same effect, likely due to the absence of active T cells in RNU nude rats in which the U87 tumour model was induced. This absence of active T cells restricts the tumour-killing effect of SDT. Nonetheless, ultrasound-assisted immune therapies such as microbubble-mediated vascular modulation, hyperthermia, thermal ablation, and SDT can trigger natural immune responses within the body^39^. However, their effectiveness may be lower in the absence of an active immune system. It is crucial to consider the immune system in cancer treatment. The distinction between the 9L and U87 models lies in the nature of the immune system. This suggests that ultrasound-triggered sonodynamic therapy could activate the immune system to help fight against the tumour and, therefore, would be most effective in immunocompetent animals. Consequently, it is imperative to investigate the mechanism through which SDT-treated tumours interact with host immunotherapeutic mechanisms. Recent research suggests that ROS can act as a regulatory agent in the immune system^40,41^. This is significant because the SDT mechanism responds to ROS production. When the innate immune response is activated, it leads to inflammation and antigenic information delivery. This causes the adaptive immune system, including B and T cells, to generate an immune response to the tumour. However, in the U87 brain tumour model, T-cell deficiency could potentially contribute to the compromised therapeutic outcome.

The SDT can induce apoptosis in brain tumours^42^, which may trigger a potential immune response in the tumour area. This immune response is crucial for effective tumour treatment^43^. In this study, the therapeutic effects were not consistent across the two rodent brain tumour models, even though the same SDT parameters were used, including the FUS sonication scheme and dose of 5-ALA. Therefore, we speculate that the immune system may play a role in SDT. For future applications of SDT combined with immunotherapy, it is essential to investigate how the immune response is altered following SDT. Immunotherapy is an emerging treatment for various types of cancers, including brain tumours. Several FDA-approved immunotherapies, such as checkpoint inhibitors and monoclonal antibodies, have been developed for this purpose. As a result, the immune system plays a crucial role in treating brain tumours. In this study, we evaluated human brain tumour U87 cells using rats that lacked an immune system. The results may only reflect the cell-killing effect resulting from the generation of ROS and not the synergistic effect that a normal immune system would provide.

Apoptosis, programmed cell death, was coupled with ROS production and mitochondrial membrane potential loss, suggesting that the dysfunction of the mitochondrial signal pathway may be involved in SDT-induced apoptosis^44^. In addition, SDT-induced tumour apoptosis has increased the expression of caspase 3 and cytochrome c in immunohistochemistry investigation. Moreover, SDT could also inhibit angiogenesis by destroying the vasculature within the tumour and thus inducing ischemia or hypoxia condition^45^.

SDT offers a therapeutic benefit in the tumour volume due to the accumulation of the sonodynamic agent (5-ALA) within the tumour. The effectiveness of the sonodynamic effect may vary among different types of tumours. As a result, in this study, we targeted an area larger than the tumour itself to ensure that we effectively sonicate both the tumour core and the surrounding regions infiltrated by the tumour.

SDT provides a promising path in conquering devastating brain tumours. There are still some bottleneck questions that need to be answered. The microbubbles-mediated FUS applications utilize acoustic signals to monitor or control the target area’s pressure^46^. Thermally ablative FUS technology uses thermometry to feedback control the ultrasound energy to create the lesion at the focal region^47^. However, the control of SDT mechanisms has not yet been explored and optimized. In this study, we demonstrated a fixed pressure to sonicate the whole brain tumour volume and had promising therapeutic results. The optimal ultrasound pressure or intensity might vary depending on the tumour’s location. Therefore, there is a need to explore the importance of precise exposure control for SDT. This will be the aim of our future studies.

## Methods

### Experiment design

Firstly, to examine the therapeutic effect of SDT in the immunocompetent brain tumour model, 9L gliosarcoma-bearing rats. Based on preliminary experiments and in order to limit the number of experiments, we selected a peak negative pressure (PNP) of 0.75 MPa (measured in water without skull attenuation). We investigated the impact of burst length on the therapeutic outcome by sonicating three SDT groups using 10 ms (*n* = 5), 50 ms (*n* = 5) and 86 ms burst lengths. Based on the results, the burst length of 86 ms was applied in the following experiments. Secondly, 9L gliosarcoma-bearing rats were randomly assigned to the following groups, and the treatments started on Day 7: (1) control (*n* = 5, no treatment), (2) three weekly treatments with FUS only (*n* = 5), (3) three weekly treatments with 5-ALA (60 mg/kg per week, *n* = 5), (4) three weekly treatments with SDT (*n* = 5). Thirdly, to investigate SDT in the immunocompromised brain tumour model, a total of twenty-two human U87 MG brain tumour-bearing nude rats were randomly assigned to the following groups and the treatments started on Day 7: (1) control (*n* = 5, no treatment), (2) three weekly treatments with FUS (86 ms burst length, *n* = 5), (3) three weekly treatments with 5-ALA (60 mg/kg per week, *n* = 6), (4) three weekly treatments with SDT (*n* = 6). Fourthly, three weekly treatments with SDT (86 ms burst length, *n* = 7) started on Day 16 for the late-stage 9L tumour treatment investigation.

### Rodent 9L gliosarcoma and human U87 MG glioma brain tumour cells

Rodent 9L gliosarcoma brain tumour cells (ATCC^®^ CRL-2200™) and human U87 MG glioblastoma (ATCC^®^ HTB-14™) purchased from ATCC were cultured respectively in DMEM (Dulbecco’s modified Eagle’s medium) and EMEM (Eagle’s Minimum Essential Medium) and supplemented with 10% heat-inactivated fetal bovine serum (FBS) in 75 cm^2^ flasks in a 5% CO_2_-containing incubator at 37 °C. Cell number and viability were calculated with an automated cell counter (LUNA™, Gyeonggi-do, South Korea) via trypan blue exclusion.

### Preparation of 5-aminolevulinic acid

The sonosensitizing agent 5-aminolevulinic acid (5-ALA, SONALA-001^®^, SonALAsense Inc., Oakland, CA, USA) was stored in the dark to prevent light at 4 °C. The agent was dissolved in phosphate-buffered saline (PBS, pH 7.4) at a 60 mg/mL concentration right before use. 5-ALA was injected over a minute via the tail vein at a 60 mg/kg dose 6 h before sonication.

### Animals and brain tumour models

Wistar rats (male, *n* = 30) with a mean weight of 311 ± 22 g and RNU nude Rats with T-cell deficiency (male, *n* = 22) with a mean weight of 260 ± 24 g were purchased from Charles River Laboratories (Wilmington, MA, USA). On a reverse light cycle, animals were housed in the Sunnybrook Research Institute animal facility (Toronto, ON, Canada) and had access to food and water *ad libitum*. All animal procedures were approved by the Animal Care Committee at Sunnybrook Research Institute and are in accordance with the Canadian Council on Animal Care and ARRIVE guidelines.

General anesthesia was induced using 5% isoflurane and then maintained at 2.5% for the tumour implantation stereotaxic surgery. Animal core body temperature was maintained between 36 and 37 °C with a feedback control heating pad. The rats were subcutaneously injected with buprenorphine (0.01–0.05 mg/kg) once daily for up to 3 days. The hair on the scalp was removed with an electric clipper. The surgical area was sterilized by scrubbing with chlorhexidine and iodine. An incision was cut on the rat’s scalp, and a burr hole was made using a drill. A total of 4 × 10^5^ 9L gliosarcoma tumour cells or U87 MG cells suspended in 10 µL of phosphate-buffered saline (PBS) and Matrigel (Corning^®^ Matrigel^®^, Discovery Labware, Inc., MA, USA) were injected into the right striatum (0.5 mm anterior and 2.8 mm lateral to the bregma at a depth of 5 mm from the dura) using a microinjection syringe (Hamilton Company, Reno, NV, USA) attached to a stereotaxic apparatus (David Kopf Instruments, Tujunga, CA, USA). After injection, the needle stayed in the brain for 5 min and was slowly withdrawn over another 1 min. The hole on the skull was covered by bone wax. The incision was then sewn up with 5 − 0 polydioxanone sutures. One week after surgery, the sutures were removed. The designed endpoint of this study was set to be either 20% body weight loss, brain tumour volume exceeding 200 mm^3^ or abnormal behaviour.

### Weekly sonodynamic treatments

**Fig. 5:**
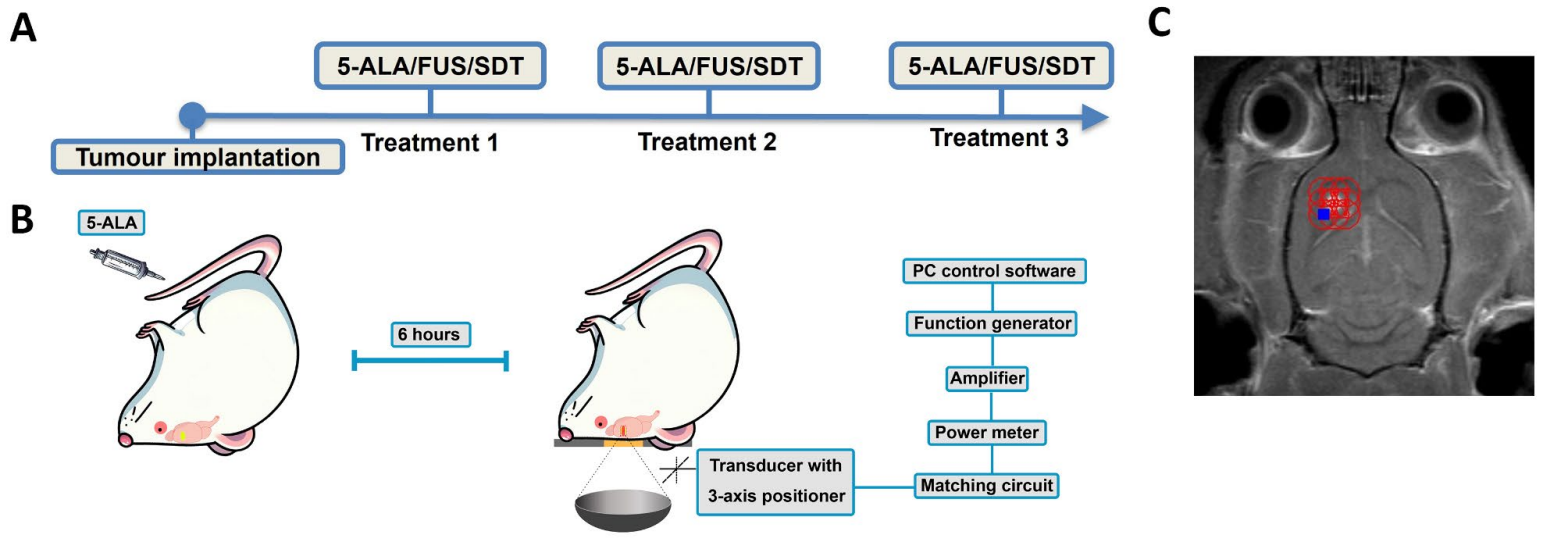
(**A**) The brain tumour models were induced using the stereotaxic injection surgery. The tumours were confirmed with MR images prior to the MR-guided FUS treatment. Three weekly treatments were performed at the designed stage. (**B**) The procedure of sonodynamic therapy. In brief, the sonodynamic agent, 5-ALA, was administered intravenously 6 h before the FUS sonication. (**C**) A representative treatment planning image was shown, and the FUS targets covering the tumour volume were placed.

A 60 mg/kg 5-ALA was slowly injected via the tail vein over 1 min, approximately six hours before focused ultrasound sonication. The hair was removed with an electrical clipper, and the hair removal cream was applied to the scalp for 5 min to remove the remaining hair further. Focused ultrasound was delivered using a pre-clinically MRI-guided FUS system (a prototype similar to LP100; FUS Inc., Toronto, ON, Canada). Pressure calibration of the transducer was performed using a fibre-optic hydrophone (Precision Acoustics Ltd., Dorset, UK). MRI spatial coordinates were co-registered to the spherically focused transducer with a focus volume of full-width-half-maximum (FWHM) 2.9 mm in diameter and 14 mm in axial. (frequency = 0.58 MHz, diameter = 75 mm, focal number = 0.8), which allows targets to be chosen from MR images. Pressure calibration for the transducer was conducted using a planar fibre optic hydrophone (active tip diameter = 10 μm; Precision Acoustics Ltd., Dorset, UK). Transducer movement within the degassed, deionized water tank was controlled with a motorized positioning system (3 degrees of freedom). For all sonications, each target received 300 bursts with a burst length of 10, 50, or 86 ms with a range of pulse repetition frequency (PRF) accordingly at a peak negative pressure (PNP) of 0.75 MPa (measured in water without skull attenuation). The total sonication period depends on the tumour volume that has to be covered. Contrast-enhanced T1-weighted images (CE-T1w) were obtained to confirm the tumour location and enable the targets of sonication to be chosen in the software. The three-weekly SDT treatment scheme is shown in Fig. 5, and the parameters are shown in Table 1.

**Table 1:** Sonodynamic therapy parameters.

	Frequency (MHz)	Pressure (MPa)	Burst length (ms)	PRF (Hz)	Target(s)	Total time (min)	Treatments	5-ALA Dose (mg/kg)
SDT 9L	0.58	0.75	10, 50, 86	1/N	Multiple	5*N	3	60
SDT U87	0.58	0.75	86	1/N	Multiple	5*N	3	60

### MR Imaging

A 7-Tesla MRI scanner (BioSpec 70/30 USR, Bruker, USA) was utilized in this study. T1-weighted contrast-enhanced (T1-CE) coronal images (TE/TR = 6 ms / 500 ms; matrix = 256 × 256; slice thickness = 1.5 mm) were used to target the tumour location. T2-weighted imaging (TE/TR = 70 ms / 4000 ms; matrix = 256 × 256; slice thickness = 1.5 mm) and T2*-weighted imaging (TE/TR = 3 ms / 800 ms; matrix = 256 × 256; slice thickness = 1.5 mm) were used to identify if there is any damage after treatment (edema and hemorrhage). During follow-up images, the animal was positioned prone, and a rat brain surface coil (T11425V3, Bruker, USA) was used to improve the image quality. MIPAV (Bethesda, MA, USA) software was used for MRI image analysis. The built-in auto-contouring function determined the tumour area, and the software calculated the tumour volume.

### Histological evaluation

The animals were sacrificed once they reached the designed endpoint. Saline perfusion was conducted intracardially and then followed by 10% formalin. Brains were placed in 10% formalin for at least 24 h before transferring to 70% ethanol. Paraffin embedding was performed for post-fixation, and the brains were cut into five µm sections for further stains. The H&E stain was used for the gross investigation of tumour morphology. Our previous study using the same sonodynamic agent^12^ conducted TUNEL and Ki67 stains to prove the cytotoxic effects after SDT. Therefore, we did not repeat the same histology examinations.

### Statistical analysis

All values are displayed as mean ± standard deviation (SD). All statistical calculations were processed on a computer using GraphPad Prism 8 (GraphPad Software, San Diego, CA, USA). The results were analyzed with a one-way analysis of variance (ANOVA) with the post-hoc Dunnett’s test, and the survival data were analyzed using a log-rank test. All *p*-values were two-sided, and a value of *p* < 0.05 was considered statistically significant in all cases.

## Data Availability

The data supporting this study’s findings are available upon reasonable request from the corresponding author.
